# Binding of SGTA to Rpn13 selectively modulates protein quality control

**DOI:** 10.1242/jcs.165209

**Published:** 2015-09-01

**Authors:** Pawel Leznicki, Jelena Korac-Prlic, Katarzyna Kliza, Koraljka Husnjak, Yvonne Nyathi, Ivan Dikic, Stephen High

**Affiliations:** 1 Faculty of Life Sciences, University of Manchester, Oxford Road, Manchester M13 9PT, UK; 2 Department of Immunology and Medical Genetics, School of Medicine, University of Split, Soltanska 2, Split 21000, Croatia; 3 Institute of Biochemistry II, School of Medicine, Goethe University, Theodor-Stern-Kai 7, Frankfurt (Main) 60590, Germany; 4 Buchmann Institute for Molecular Life Sciences, School of Medicine, Goethe University, Theodor-Stern-Kai 7, Frankfurt (Main) 60590, Germany

**Keywords:** Bag6, Mislocalised proteins, Proteasomes, Protein degradation, TPR, Ubiquitylation

## Abstract

Rpn13 is an intrinsic ubiquitin receptor of the 26S proteasome regulatory subunit that facilitates substrate capture prior to degradation. Here we show that the C-terminal region of Rpn13 binds to the tetratricopeptide repeat (TPR) domain of SGTA, a cytosolic factor implicated in the quality control of mislocalised membrane proteins (MLPs). The overexpression of SGTA results in a substantial increase in steady-state MLP levels, consistent with an effect on proteasomal degradation. However, this effect is strongly dependent upon the interaction of SGTA with the proteasomal component Rpn13. Hence, overexpression of the SGTA-binding region of Rpn13 or point mutations within the SGTA TPR domain both inhibit SGTA binding to the proteasome and substantially reduce MLP levels. These findings suggest that SGTA can regulate the access of MLPs to the proteolytic core of the proteasome, implying that a protein quality control cycle that involves SGTA and the BAG6 complex can operate at the 19S regulatory particle. We speculate that the binding of SGTA to Rpn13 enables specific polypeptides to escape proteasomal degradation and/or selectively modulates substrate degradation.

## Abstract (precis)

**Highlighted Article:** Binding of SGTA to the proteasome delays substrate degradation, thereby providing a mechanism for potentially viable proteins to be rescued for reuse.

## INTRODUCTION

The ubiquitin-proteasome system (UPS) constitutes a main pathway for protein degradation in eukaryotic cells, with polypeptides destined for disposal via this route bearing ubiquitin chains. The selective and covalent attachment of the small ubiquitin polypeptide to these proteins is typically through lysine residues within the substrates and occurs through a cascade of sequential reactions catalysed by E1, E2 and E3 enzymes (Komander and Rape, 2012). Furthermore, ubiquitin itself contains seven lysine residues, each of which can also serve as acceptor sites during ubiquitylation, leading to the formation of polyubiquitin chains with different linkages (Komander and Rape, 2012). Amongst these, K48- and K11-linked chains typically serve to hallmark proteins for proteasomal degradation (Komander and Rape, 2012). The 26S proteasome is a multiprotein complex composed of a 20S catalytic core where proteolysis occurs, and a 19S regulatory particle that controls substrate entry (Bhattacharyya et al., 2014; Komander and Rape, 2012). Protein ubiquitylation can be reversed by the action of proteases that are collectively known as deubiquitylating enzymes (DUBs) (Komander and Rape, 2012; Komander et al., 2009), and the removal of polyubiquitin by proteasomal DUBs precedes substrate degradation at the catalytic core (Bhattacharyya et al., 2014; Komander and Rape, 2012; Wauer and Komander, 2014).

Substrate delivery to the proteasome is facilitated by both intrinsic (such as Rpn10 and Rpn13) (Husnjak et al., 2008; van Nocker et al., 1996) and shuttle (such as Rad23 and ubiquilins) ubiquitin receptors (Wang and Terpstra, 2013). Shuttle ubiquitin receptors bind ubiquitylated proteins through ubiquitin-associated domains (UBAs) and simultaneously interact with the proteasome through their ubiquitin-like domains (UBLs) (Wang and Terpstra, 2013). Rpn10 and Rpn13 interact with the UBLs of such shuttle factors, but can also bind directly to ubiquitylated substrates (Bhattacharyya et al., 2014; Husnjak and Dikic, 2012; Husnjak et al., 2008; van Nocker et al., 1996). It has been suggested that effective proteasomal degradation requires simultaneous recognition of the polyubiquitylated substrate by both the Rpn10 and Rpn13 subunits of the 19S regulatory particle (Bhattacharyya et al., 2014; Kang et al., 2006; Komander and Rape, 2012; Sakata et al., 2012). Substrates are subsequently deubiquitylated by Rpn11, a 19S-localised DUB, helping to maintain the cellular pool of free ubiquitin available for conjugation (Bhattacharyya et al., 2014; Komander and Rape, 2012; Komander et al., 2009; Wauer and Komander, 2014). Two additional DUBs, USP14 and UCHL5 (also known as and hereafter referred to as UCH37), also associate with the proteasome, although their precise roles are unclear (D'Arcy and Linder, 2012; Komander and Rape, 2012; Lee et al., 2011). Hence, whereas Rpn11 removes ubiquitin chains from proteasomal substrates en bloc USP14 and UCH37 seem to preferentially cleave off distal ubiquitin moieties, suggesting that they provide an editing or quality control function that can rescue inefficiently or prematurely ubiquitylated polypeptides (Bhattacharyya et al., 2014; D'Arcy and Linder, 2012; Lee et al., 2011). The recruitment of UCH37 to the proteasome is mediated by the C-terminal region of Rpn13, indicating that substrate recognition and ubiquitin-chain processing might be coupled (see D'Arcy and Linder, 2012; Komander et al., 2009 and references therein).

The UPS plays a central role in protein quality control, providing one of the primary routes by which the cell can remove potentially deleterious, aberrant and misfolded proteins, and maintain cellular protein homeostasis (Bhattacharyya et al., 2014; Buchberger et al., 2010; Komander and Rape, 2012; Wang and Terpstra, 2013; Wauer and Komander, 2014). A variety of effectors operate upstream of the UPS acting to recognise different classes of defective proteins and regulate their selective removal (Buchberger et al., 2010; Wang and Terpstra, 2013). Two such effectors are small glutamine-rich tetratricopeptide repeat containing protein alpha (SGTA) and the heterotrimeric BAG6 complex that, together, deal with polypeptide substrates that inappropriately expose hydrophobicity to the cytosol (Hessa et al., 2011; Leznicki and High, 2012; Minami et al., 2010; Rodrigo-Brenni et al., 2014; Wunderley et al., 2014). Hence, SGTA and the BAG6 complex are implicated in the quality control of mislocalised and secretory proteins, collectively termed mislocalised proteins (MLPs), which have failed to be correctly delivered to the endoplasmic reticulum (ER) and, consequently, become localised to the cytosol (Hessa et al., 2011; Leznicki and High, 2012; Rodrigo-Brenni et al., 2014; Wunderley et al., 2014).

Current models suggest that BAG6 and SGTA can each recognise a range of hydrophobic substrates located in the cytosol and direct them to an appropriate biosynthetic or degradative route. Hence, tail-anchored membrane proteins that follow a post-translational pathway for membrane insertion encounter both SGTA and the BAG6 complex prior to their TRC40-dependent integration at the ER (Leznicki et al., 2010; Mariappan et al., 2010; Mock et al., 2015). By contrast, the normal fate of hydrophobic substrates that are unable to translocate into or across the ER membrane is rapid, BAG6-facilitated, proteasomal degradation (Hessa et al., 2011; Rodrigo-Brenni et al., 2014; Wunderley et al., 2014). Both overexpression and knockdown studies indicate that SGTA antagonises the actions of the BAG6 complex to delay the proteasomal degradation of MLPs (Leznicki and High, 2012; Wunderley et al., 2014). Furthermore, the N-terminal domain of an SGTA homodimer can bind to the BAG6 complex through its two ubiquitin-like domain (UBL)-containing subunits, providing a physical link between these two quality control factors (Chartron et al., 2012; Darby et al., 2014; Leznicki et al., 2013; Xu et al., 2012). In the context of proteasomal degradation, BAG6 facilitates the RNF126-dependent ubiquitylation and proteasomal degradation of MLPs (Hessa et al., 2011; Rodrigo-Brenni et al., 2014), whereas SGTA acts to inhibit MLP degradation, most probably by favouring their deubiquitylation (Leznicki and High, 2012; Wunderley et al., 2014). In a physiological context it has been suggested that the SGTA-dependent antagonisation of BAG6 provides a rescue pathway for potentially viable substrates – such as tail-anchored proteins – that are prematurely ubiquitylated (Leznicki and High, 2012; Wunderley et al., 2014). Alternatively, cycles of substrate ubiquitylation and deubiquitylation might normally help to facilitate the selective degradation of MLPs (Brodsky, 2013; Zhang et al., 2013).

In seeking so-far-unknown functions for the intrinsic proteasomal ubiquitin receptor Rpn13, we identified a novel interaction with SGTA. This interaction was initially suggested by the results of a yeast two-hybrid screen and, subsequently, validated using two different pull-down strategies, which showed that the C-terminal region of Rpn13 binds to the central tetratricopeptide repeat (TPR) domain of SGTA. Since Rpn13 acts as a proteasomal ubiquitin receptor, we speculated that SGTA influences substrate access to the proteasome. To test this hypothesis, we investigated the potential role of the Rpn13–SGTA interaction in the proteasomal degradation of MLPs, a process previously shown to be regulated by both SGTA (Leznicki and High, 2012; Wunderley et al., 2014) and its interacting partner BAG6 (Hessa et al., 2011; Rodrigo-Brenni et al., 2014). We show that the binding of exogenous SGTA to Rpn13 results in a substantial increase in the steady-state level of MLPs. Inhibiting this interaction by overexpression of the Rpn13 C-terminal region or mutation of the SGTA TPR region negates the effect of SGTA overexpression on MLP levels. These data support a model whereby SGTA and its interacting partner the BAG6 complex can influence the fate of MLPs at the proteasome. We speculate that these components modulate the access of such substrates to the proteasome through their respective partners Rpn13 (this study) and Rpn10 (Kikukawa et al., 2005; Minami et al., 2010). This model suggests that SGTA and BAG6 control the fate of MLPs even after their arrival at the proteasome, and provides the basis for a potential substrate rescue pathway and/or a mechanism to enhance the selectivity of substrate degradation.

## RESULTS

### Rpn13 interacts with SGTA

**Fig. 1.**
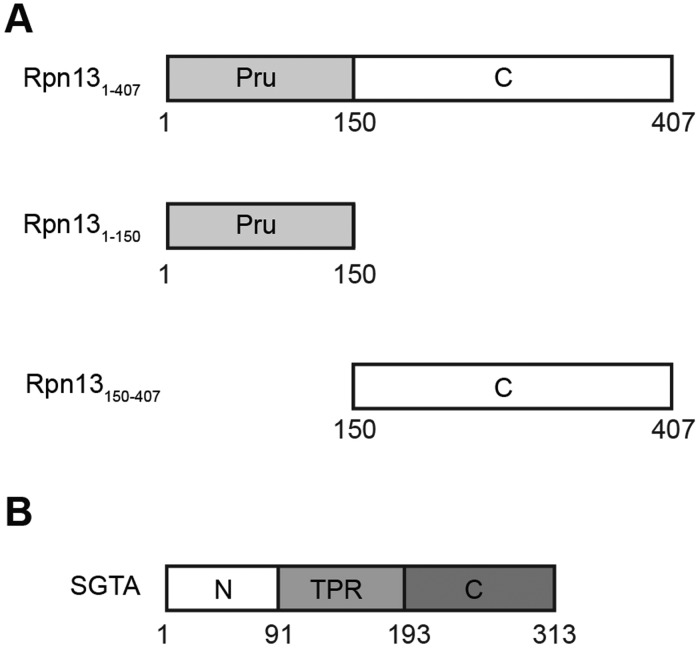
**Domain organisation of Rpn13 and SGTA.** (A,B) The discrete domains present in Rpn13 (A) and SGTA (B) are indicated together with the fragments of Rpn13 used for yeast two-hybrid analysis. For A: Pru, pleckstrin-like receptor for ubiquitin, C, C-terminal region of Rpn13 that binds UCH37. For B: N, N-terminal region responsible for both homo-oligomerisation and the binding of ubiquitin-like domains (UBLs); TPR, tetratricopeptide-repeat-containing domain; C, C-terminal glutamine-rich region implicated in binding to hydrophobic substrates.

**Fig. 2.**
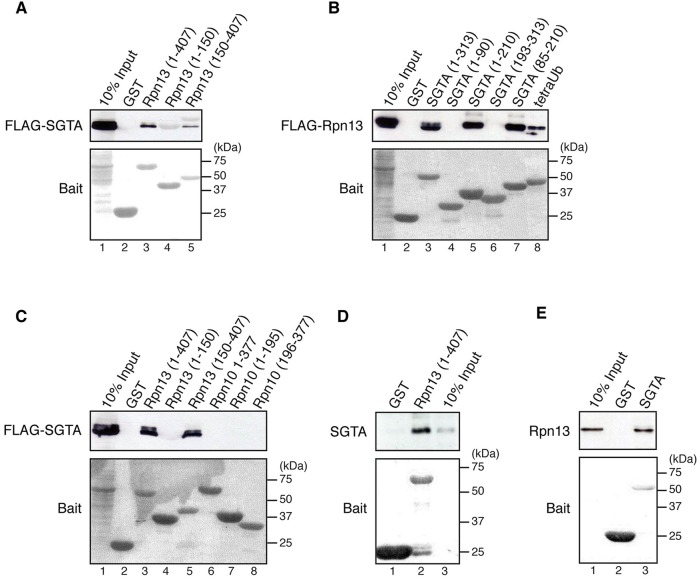
**SGTA interacts with Rpn13 *in vivo* and *in vitro.*** (A–C) HeLa cells were transiently transfected with NpFLAG-CMV2-SGTA (A,C) or NpFLAG-CMV2-mouse Rpn13 (B), lysed 24 h post-transfection and the soluble fraction incubated with GST-tagged proteins immobilised on Glutathione Sepharose beads as indicated. Bound proteins were detected by western blotting with anti-FLAG antibody, whereas GST-tagged proteins were visualised by staining the membranes with Ponceau S (see panel labelled Bait). GST-tagged tetraubiquitin (tetraUb) was used as a positive control for FLAG-Rpn13 binding. (D,E) Approximately 5 µg of purified recombinant SGTA (D) or Rpn13 (E) was incubated with equivalent amounts of indicated purified GST-tagged proteins immobilised on Glutathione Sepharose beads and their binding followed by western blotting with anti-SGTA or anti-Rpn13 antibodies, respectively. Immobilised GST-tagged proteins were visualised by Ponceau S staining of the membranes (Bait).

To better understand the role of Rpn13 during proteasomal degradation, we used full-length mouse Rpn13 (Rpn13_1-407_), as well as its N-terminal pleckstrin-like receptor for ubiquitin (Pru) domain (amino acid residues 1–150; hereafter referred to as Rpn13_1-150_) and distinct C-terminal region (amino acid residues 150–407; hereafter referred to as Rpn13_150-407_) (Fig. 1A), as baits in yeast two-hybrid screens with a thymus cDNA library prey. This approach identified SGTA as a potential interacting partner of Rpn13_150-407_ (data not shown). To validate the yeast two-hybrid data, purified GST-tagged Rpn13, Rpn13_1-150_ and Rpn13_150-407_, were used as baits in pull-down experiments primed with lysate from HeLa cells overexpressing FLAG-tagged SGTA. This showed a specific physical interaction of full-length Rpn13_1-407_ and the Rpn13_150-407_ fragment with exogenous FLAG-SGTA (Fig. 2A, lanes 3–5). To map the Rpn13-binding site on SGTA, a variety of SGTA deletion mutants were purified as recombinant GST fusion proteins and their interaction with exogenous FLAG-tagged Rpn13 present in HeLa cell lysate was examined. This approach identified the central region of SGTA, comprising residues 85-210, as necessary and sufficient for Rpn13 binding (Fig. 2B, cf. lanes 3–7). This corresponds to the central tetratricopeptide repeat (TPR) domain of SGTA (Fig. 1B), a region previously implicated in binding both molecular chaperones and viral proteins (Dutta and Tan, 2008; Fielding et al., 2006; Liou and Wang, 2005; Walczak et al., 2014). In contrast to its interaction with Rpn13, FLAG-SGTA did not bind to purified human Rpn10 or its fragments (Fig. 2C, lanes 6–8), confirming the specificity of its interaction with the Rpn13 ubiquitin receptor (Fig. 2C, cf. lanes 3–5). Since HeLa lysate is most likely to contain a number of endogenous SGTA and/or Rpn13-binding partners, we further tested the nature of the interaction by using recombinant Rpn13 and SGTA. The SGTA–Rpn13 interaction could be recapitulated using purified proteins (Fig. 2D and E), and we concluded that the two components bind directly to each other.

Previous studies have shown that all cellular Rpn13 is incorporated into the proteasome at steady state (Hamazaki et al., 2006; Qiu et al., 2006), and we speculated that, in a cellular context, the newly defined Rpn13–SGTA interaction mediates the proteasomal recruitment of SGTA. To test this hypothesis, we first purified proteasomes by using the HEK293^Rpn11-HTBH^ cell line that constitutively expresses a tagged form of the Rpn11 subunit in addition to the endogenous protein (Wang et al., 2007). This cell line provides a convenient approach to isolate native proteasomes and has been used in several studies (Chen et al., 2010; Tsimokha et al., 2014; Wang et al., 2010). We found that a small fraction of endogenous SGTA was recovered with intact proteasomes following their isolation by using a streptavidin pull down (supplementary material Fig. S1, lanes 1–4). The association of endogenous SGTA with the proteasome was most apparent when cells had been pre-treated with the proteasome inhibitor MG132 prior to purification (supplementary material Fig. S1, cf. lanes 3 and 4). On the basis of these data, we concluded that Rpn13 provides a binding site for SGTA at the proteasome, and we next explored the functional consequences of this interaction. Whereas the increase in proteasomal SGTA observed upon treatment with MG132 was consistent with the stabilisation of a direct interaction with Rpn13 (cf. Fig. 2), we cannot rule out the alternative possibility that SGTA also binds to Rpn13 through ubiquitylated substrates that accumulate on the proteasome in the presence of the inhibitor (Isakov and Stanhill, 2011).

### SGTA promotes the proteasomal association of MLPs

**Fig. 3.**
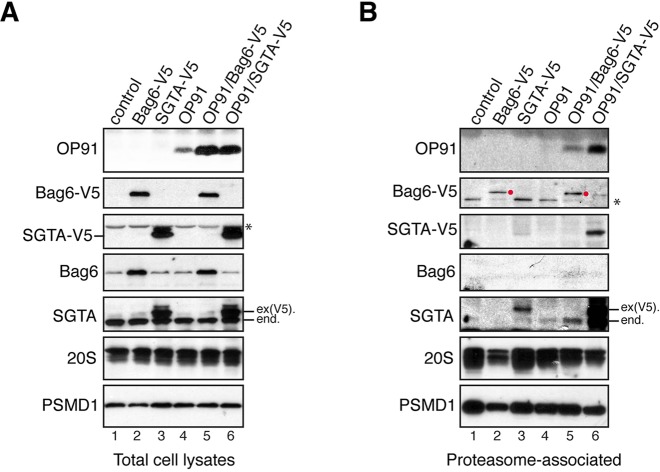
**OP91 and SGTA co-purify with the proteasome when co-expressed.** (A,B) HEK293^Rpn11-HTBH^ cells were transiently transfected with plasmids encoding the indicated proteins (lanes 2–6) or an empty vector control (lanes 1). Total cell lysates (A) and proteasomal fractions isolated by using streptavidin beads (B), and were analysed by western blotting with appropriate antibodies for the presence of the MLP substrate, OP91, exogenous Bag6-V5, exogenous SGTA-V5, endogenous Bag6 and endogenous SGTA. Proteasomal recovery was confirmed by using antibodies against subunits of the 20S (20S), and 19S (PSMD1) proteasome as indicated (see also supplementary material Fig. S1). Endogenous (end.) and exogenous [ex(V5).] SGTA are identified, as is overexpressed Bag6-V5 recovered with the proteasome (red circles). *, Non-specific, crossreacting species (see also supplementary material Fig. S2).

The capacity of both SGTA and the Bag6 subunit of the heterotrimeric BAG6 complex to bind hydrophobic polypeptides (Hessa et al., 2011; Leznicki et al., 2013, 2011; Minami et al., 2010; Wunderley et al., 2014; Xu et al., 2012), and the proteasome (this study; Kikukawa et al., 2005), raised the possibility that these components modulate the fate of MLPs at the proteasome. To address this question, SGTA and Bag6 were transiently overexpressed in HEK293^Rpn11-HTBH^ cells together with OP91, an N-terminal fragment of the polytopic membrane protein opsin that acts as an MLP (Wunderley et al., 2014). SGTA co-expression led to a marked increase in steady-state OP91 in both HEK293^Rpn11-HTBH^ cells (Fig. 3A, OP91 panel, cf. lanes 4 and 6) and the parental line (supplementary material Fig. S2A), consistent with previous studies (Leznicki and High, 2012; Wunderley et al., 2014). Interestingly, overexpression of the Bag6 protein had a similar effect on the level of OP91 (Fig. 3A, OP91 panel, lanes 4 and 5), in agreement with the previously reported dominant-negative effect exogenous Bag6 expression has on the degradation of aberrant membrane proteins (Payapilly and High, 2014).

No proteasome-associated OP91 was apparent with the parental cell line (supplementary material Fig. S2B, cf. OP91 panel). Likewise, when intact proteasomes were isolated from HEK293^Rpn11-HTBH^ cells, OP91 was undetectable in the absence of additional factors (Fig. 3B, OP91 panel, lane 4). By contrast, OP91 was readily detectable when the proteasomal fraction was isolated from cells that co-express exogenous SGTA (Fig. 3B, OP91 panel, cf. lanes 4 and 6). Notably, the proteasomal association of this MLP substrate was mirrored by the recruitment of both exogenous and endogenous SGTA to the proteasome [Fig. 3B, SGTA-V5 (exogenous SGTA) and SGTA panels, cf. lanes 3, 4 and 6]. By contrast, although Bag6 co-expression led to a comparable increase in steady-state levels of OP91 (Fig. 3A, OP91 panel, cf. lanes 4–6), the amount of proteasome-associated OP91 was much lower (Fig. 3B, OP91 panel, cf. lanes 4–6). Likewise, the recovery of exogenous Bag6 with the proteasome appeared unaffected by OP91 co-expression (Fig. 3B, Bag6-V5 panel, cf. lanes 2 and 5, red circles). Interestingly, Bag6 co-expression with OP91 did appear to enhance the proteasomal recruitment of endogenous SGTA (Fig. 3B, SGTA panel, cf. lanes 2, 4 and 5, see component labelled ‘end.’). In short, the enhanced steady-state MLP levels observed upon SGTA overexpression correlate with a specific increase in the binding of both OP91 and SGTA to the proteasome. Given that SGTA is known to bind a variety of hydrophobic substrates, including MLPs and tail-anchored membrane proteins (Leznicki et al., 2010, 2011; Liou and Wang, 2005; Wunderley et al., 2014), we conclude that proteasome-associated SGTA might influence MLP stability by regulating the access of such substrates to the catalytic core. To test this hypothesis, we explored the outcome of perturbing the Rpn13-dependent binding of exogenous SGTA to the proteasome.

### SGTA binding to Rpn13 regulates MLP stability

**Fig. 4.**
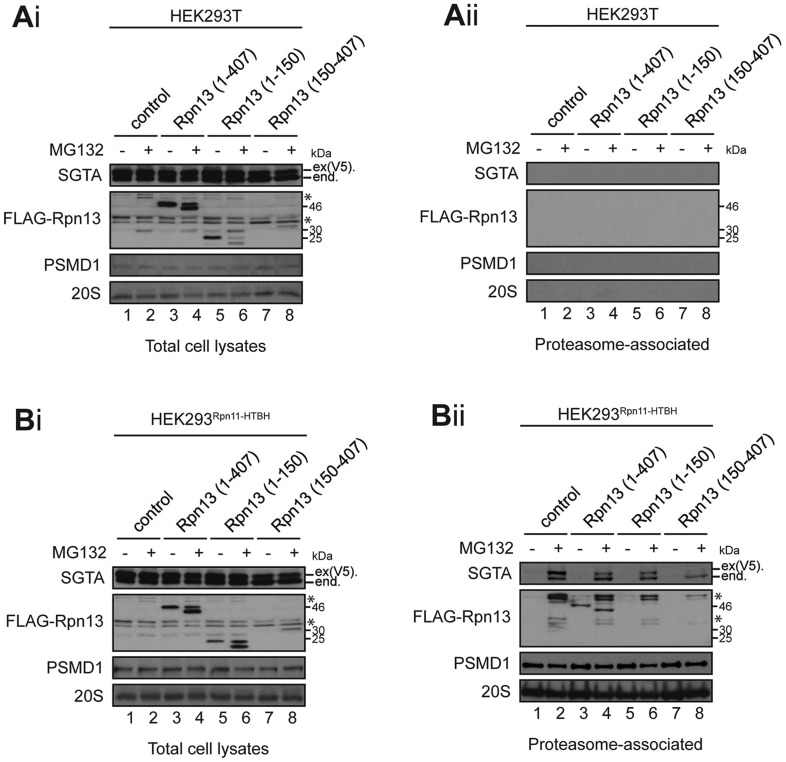
**The Rpn13 C-terminal region inhibits binding of SGTA to the proteasome.** (Ai–Bii) Parental HEK293T cells (Ai and Aii) or HEK293^Rpn11-HTBH^ cells that express an exogenous tagged form of Rpn11 (Bi and Bii) were transiently co-transfected with pcDNA5-SGTA-V5 and empty NpFLAG-CMV2 plasmid (lanes 1 and 2) or NpFLAG-CMV2 encoding the indicated variants of Rpn13 (lanes 3–8). Cells were treated as indicated with 10 µM MG132 or DMSO (solvent control) for 16 h, and then total cell lysates (Ai,Bi), or proteasomal fractions isolated under native conditions using streptavidin beads (Aii,Bii) were prepared. The samples were analysed for endogenous (end.) and exogenous [ex(V5).] SGTA and FLAG-Rpn13 variants (FLAG-Rpn13) by western blotting. Proteasomal recovery was confirmed by western blotting for 20S components and PMSD1 (cf. Fig. 3). *, Non-specific, crossreacting, species detected by certain antibodies.

**Fig. 5.**
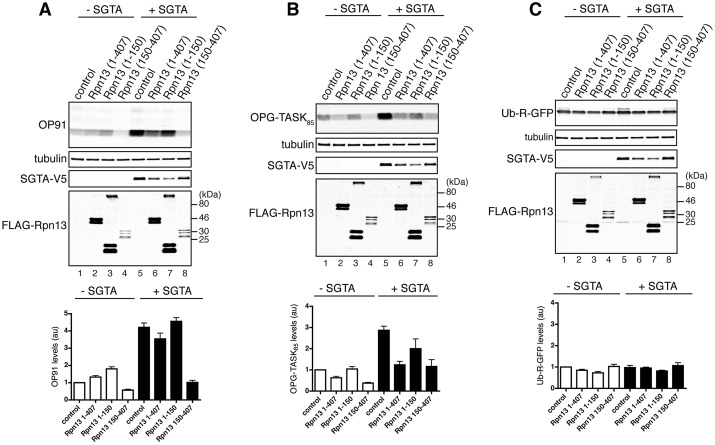
**Exogenous Rpn13 reverses SGTA-mediated increase in MLP levels.** (A–C) HeLa cells were co-transfected with plasmids encoding OP91 (A), OPG-TASK_85_ (B) or Ub-R-GFP (C), together with pcDNA5-SGTA-V5, or a control vector, and NpFLAG-CMV2 encoding the indicated variants of Rpn13 or empty NpFLAG-CMV2 plasmid as indicated. Substrate levels were examined 22 h post-transfection by quantitative western blotting of total cell lysate, the resulting signals normalised to tubulin and plotted relative to the amount of substrate in control cells (lane 1 in each panel). The values show standard errors; *n*≥3. Overexpressed exogenous SGTA and Rpn13 were visualised by western blotting with antibodies recognising the V5 or FLAG tags.

Our data show that SGTA binds to a C-terminal region of Rpn13, Rpn13_150-407_, that is distinct from the N-terminal Pru domain, which interacts with the proteasome and ubiquitin (Chen et al., 2010; Husnjak et al., 2008; Schreiner et al., 2008). On this basis, we speculated that overexpression of SGTA increases its occupancy of Rpn13, thereby antagonising the proteasomal degradation of MLPs and resulting in an increase of their steady-state expression level (Fig. 3A; see also Wunderley et al., 2014). To test this hypothesis, we investigated the effect of Rpn13 overexpression on the association of exogenous SGTA with proteasomes (cf. Fig. 3; supplementary material Figs S1 and S2). Although exogenous V5-tagged SGTA was expressed under all conditions tested (Fig. 4Ai and Bi, see SGTA panel), it was only detected in the proteasome-associated fraction recovered from cells expressing HTBH-tagged Rpn11 after treatment with MG132 [Fig. 4Aii and Bii, SGTA panel, product labelled ex(V5).]. Notably, the amounts of exogenous SGTA and its endogenous counterpart that were recovered with the proteasome are both reduced upon Rpn13_150-407_ co-expression (Fig. 4Bii, SGTA panel, lanes 2 and 8). By contrast, although present at higher levels (Fig. 4Ai and Bi, FLAG-Rpn13 panel; see also Fig. 5), overexpression of full-length Rpn13, or of its N-terminal fragment, have far less of an effect on the proteasomal association of SGTA (Fig. 4Bii, SGTA panel, lanes 2, 4, 6 and 8). On this basis, we conclude that – when overexpressed – the C-terminal Rpn13_150-407_ fragment can compete for binding to available SGTA, thereby reducing its association with the proteasome.

We next asked whether a reduction of the proteasome-associated fraction of exogenous SGTA by overexpressing Rpn13_150-407_ has any impact on the fate of OP91 (see Fig. 3) and an alternative MLP OPG-TASK_85_ that is derived from the K^+^-channel TASK-1 (Wunderley et al., 2014). Co-expression of Rpn13_150-407_ and the MLPs alone led to a reduction in detectable amounts of OP91and OPG-TASK_85_ (Fig. 5A and B, lanes 1 and 4, see also accompanying graphs); suggesting that the Rpn13_150-407_-mediated displacement of endogenous SGTA (Fig. 4Bii) reduces steady-state levels of MLP. The effect of Rpn13_150-407_ co-expression is much more striking when exogenous SGTA is present, with steady-state levels of MLP approaching those seen without exogenous SGTA (Fig. 5A and B, cf. lanes 1, 5 and 8; see also accompanying graphs). Co-expressing full-length Rpn13 with exogenous SGTA had an effect that is comparable with that of Rpn_13150-407_ for the MLP substrate OPG-TASK_85_, but is more-modest in the case of OP91 (Fig. 5A and B, lanes 5–8). By contrast, co-expression of the N-terminal Rpn13_1-150_ domain in combination with SGTA has relatively little effect (Fig. 5A and B, cf. lanes 5, 7 and 8). The substrate specificity of these effects was explored by using ubiquitin–arginine–GFP (Ub-R-GFP) (Dantuma et al., 2000), a proteasomal N-end rule substrate that appears to be insensitive to changes in SGTA levels (Leznicki and High, 2012; Wunderley et al., 2014). Despite comparable levels of expression of SGTA and all three Rpn13 variants (Fig. 5A–C), steady-state levels of Ub-R-GFP were essentially unaltered by any of the combinations tested (Fig. 5C, lanes 1–8; and accompanying graph). On the basis of these results, we conclude that the ability of exogenous SGTA to enhance steady-state MLP levels is strongly dependent upon its binding to the C-terminal region of Rpn13.

**Fig. 6.**
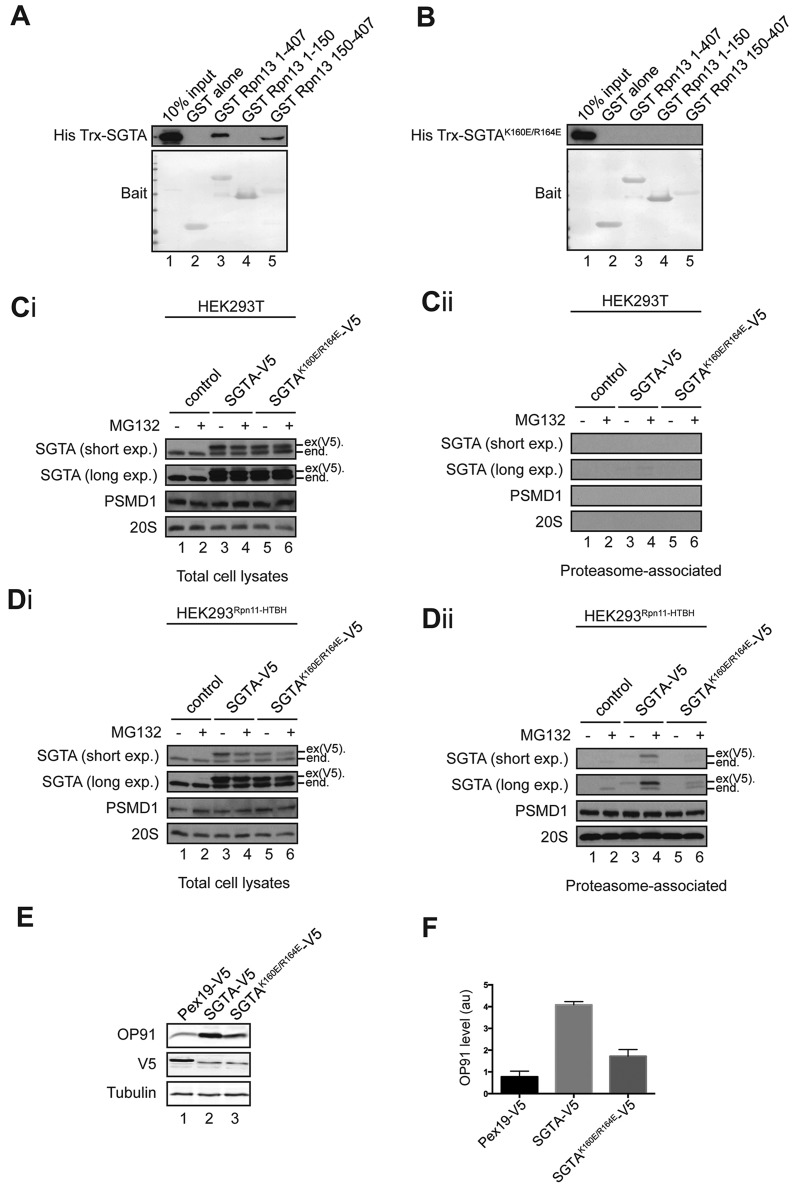
**An SGTA mutant that is impaired in proteasome binding is also defective in enhancing MLP levels.** (A,B) Approximately 5 µg of purified recombinant His Trx-SGTA (A) or His Trx-SGTA K160E/R164E (B) was incubated with equivalent amounts of immobilised GST (lane 1) or immobilised GST-tagged Rpn13 derivatives (lanes 2–5), and bound recombinant SGTA was visualised by western blotting with an anti-His tag antibody. Immobilised GST-tagged proteins were visualised by Ponceau S staining of the membranes (Bait). (Ci–Dii) Parental HEK293T cells (Ci, Cii), or HEK293^Rpn11-HTBH^ cells (Di, Dii), were transiently transfected with a control vector (lanes 1 and 2), or plasmids encoding V5 tagged SGTA variants (lanes 3–6), as indicated. Treatment with MG132, and sample processing and analysis were as described for Fig. 4. Endogenous (end.) and exogenous [ex(V5).] SGTA were detected by western blotting, and both short and long exposures of the resulting enhanced chemiluminescence signals are shown. (E) HeLa Flp-In T-REx cells overexpressing OP91 under an inducible promoter were transiently transfected with either a control plasmid (Pex19-V5, lane 1) or plasmids encoding V5-tagged SGTA variants as indicated (lanes 2 and 3), grown overnight, and then induced to express OP91. Cells were harvested the following day and OP91 levels were determined by quantitative western blotting of total cell lysate. (F) The resulting OP91 signals were normalised to tubulin and expressed relative to the amount of substrate in control cells (lane 1) with standard errors for *n*=3. Overexpressed exogenous Pex19 and SGTA variants were also visualised by western blotting for their V5-tag.

Given that the central TPR domain of SGTA (Fig. 1B) is responsible for binding Rpn13 (see Fig. 2B), we speculated that this interaction is sensitive to point mutations that perturb the binding of other components to this region (Walczak et al., 2014). Hence, a previously defined K160E/R164E mutant version of SGTA that is defective in binding to Hsc70 was created (Walczak et al., 2014; Xu et al., 2012) and its ability to bind Rpn13 tested. Whereas a recombinant fusion protein containing wild-type SGTA (Leznicki and High, 2012) bound to both full-length Rpn13 and its C-terminal domain (Fig. 6A, His Trx-SGTA panel), no interaction with the K160E/R164E variant was detected (Fig. 6B, His Trx-SGTA panel). Likewise, when equivalent versions of these SGTA variants were expressed in parental HEK293T or HEK293^Rpn11-HTBH^ cells (Fig. 6Ci and Di), the amount of the SGTA K160E/R164E-V5 mutant recovered with the proteasome was substantially reduced [Fig. 6Dii, SGTA panels, lanes 3–6, see product labelled ex(V5).]. On this basis, we conclude that the SGTA K160E/R164E mutant is defective in its Rpn13-mediated association with the proteasome. Finally, we used the SGTA K160E/R164E mutant as an alternative tool to test the contribution of the proteasomal binding of SGTA to its role in MLP quality control. Once again (Figs 3 and 5; see also Wunderley et al., 2014), overexpression of SGTA-V5 led to a substantial increase in OP91 (Fig. 6E, OP91 panel, cf. lanes 1 and 2), with a four-fold increase in its steady-state level (Fig. 6F). Strikingly, although both versions of SGTA-V5 were expressed at the same level (Fig. 6E, V5 panel, lanes 2 and 3), the K160E/R164E mutant was far less effective at enhancing the steady-state level of OP91 (Fig. 6E, OP91 panel, lanes 2 and 3; Fig. 6F). We, therefore, conclude that the binding of SGTA to the Rpn13 subunit of the proteasome makes an important contribution to the role of SGTA during the quality control of MLPs.

## DISCUSSION

Hydrophobic MLPs enter a cytosolic quality control pathway that appears to be mediated by the coordinated activity of the BAG6 complex and SGTA (Hessa et al., 2011; Leznicki and High, 2012). Hence, BAG6 promotes the RNF126-dependent ubiquitylation and proteasomal degradation of MLPs (Hessa et al., 2011; Rodrigo-Brenni et al., 2014), whereas SGTA acts to delay their degradation and promotes the accumulation of deubiquitylated MLPs (Leznicki and High, 2012; Wunderley et al., 2014). We now show that SGTA is selectively recruited to the proteasome through Rpn13, and provide evidence that this interaction may regulate MLP stability. Using yeast two-hybrid and biochemical approaches, we identify a direct physical interaction between the TPR region of SGTA and the C-terminal region of Rpn13. Treatment with MG132 results in a fraction of endogenous SGTA becoming stably associated with the proteasome, consistent with our proposal that these components interact in a cellular context.

**Fig. 7.**
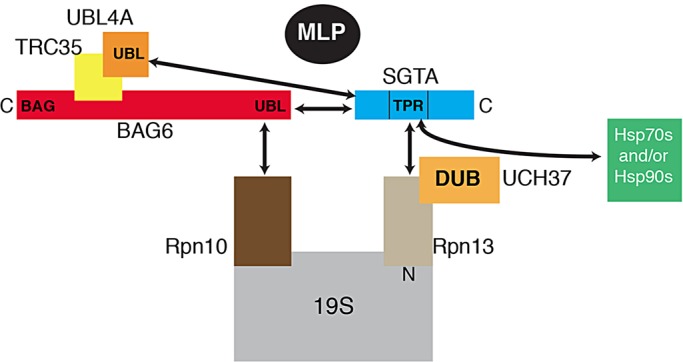
**Model describing the modulation of MLP quality control at the proteasome by BAG6 and SGTA.** The heterotrimeric BAG6 complex, composed of Bag6, TRC35 and UBL4A, recognises cytosolic MLPs and stimulates their ubiquitylation. It might also contribute to substrate delivery to the proteasome following the interaction of the Bag6 subunit with Rpn10 (Kikukawa et al., 2005; Minami et al., 2010). SGTA is recruited to the C-terminal region of Rpn13 through its central TPR domain (this study), in concert with MLPs that might bind to SGTA and/or the Pru domain of Rpn13, depending on their ubiquitylation status. The proteasome-associated deubiquitylase UCH37 also binds Rpn13 (Bhattacharyya et al., 2014; Hamazaki et al., 2006; Yao et al., 2006), providing a potential molecular basis for a putative ‘rescue pathway’ that facilitates the deubiquitylation of previously modified MLPs (Leznicki and High, 2012; Wunderley et al., 2014). The proximity of the Rpn10 and Rpn13 subunits (Bhattacharyya et al., 2014) is consistent with the suggestion that, following delivery to the proteasome, substrates undergo cycles of ubiquitylation and deubiquitylation in response to the respective actions of the BAG6 complex and SGTA (Hessa et al., 2011; Leznicki and High, 2012; Rodrigo-Brenni et al., 2014; Wunderley et al., 2014). The Hsp70 and Hsp90 molecular chaperones could also contribute to this hypothetical quality-control process (Liou and Wang, 2005; Walczak et al., 2014).

Our previous studies indicated that SGTA antagonises the BAG6-dependent ubiquitylation and proteasomal degradation of MLPs, a role that is particularly apparent when cellular SGTA levels are increased following its overexpression (Leznicki and High, 2012; Wunderley et al., 2014). We now offer a molecular basis for this effect by providing evidence that SGTA can regulate the access of MLPs to the proteasomal core in a manner that leaves other substrates, as exemplified by Ub-R-GFP, unaffected. Thus, the increased MLP levels observed upon SGTA overexpression correlate with an increase in the association of the MLP, and both exogenous and endogenous SGTA, with the proteasome. Similar to the effect of an SGTA knockdown (Wunderley et al., 2014), co-expression of an Rpn13 C-terminal region reduces steady-state MLP levels, and our data are consistent with a partial displacement of endogenous SGTA from the proteasome. This effect of Rpn13 is even more striking in the context of SGTA overexpression, which normally stabilises MLPs leading to their non-physiological accumulation (Wunderley et al., 2014). Under these circumstances, Rpn13_150-407_ co-expression substantially reduces the binding of exogenous SGTA to the proteasome and reverses the SGTA mediated increase in steady-state MLP levels. These findings are consistent with a model in which Rpn13-bound SGTA binds to MLPs and delays their proteasomal degradation (see Fig. 7; cf. Wunderley et al., 2014).

The significance of the interaction between SGTA and the proteasome during the quality control of MLPs is further supported by an SGTA variant with an altered TPR region (cf. Walczak et al., 2014). Hence, the loss of proteasome binding observed with the SGTA K160E/R164E mutant is accompanied by a substantial reduction in its effectiveness at enhancing steady-state MLP levels. Like Rpn13, Rpn10 is also an intrinsic ubiquitin receptor of the proteasome (Bhattacharyya et al., 2014; van Nocker et al., 1996), and it has previously been reported to bind to Bag6 (Kikukawa et al., 2005; Minami et al., 2010). Interestingly, the overexpression of Rpn10 leads to a modest increase in steady-state MLP levels, and this effect appears to be cumulative with the more pronounced effect of SGTA overexpression (supplementary material Fig. S3). It has been suggested that a single polyubiquitin chain can be captured by both the Rpn10 and Rpn13 ubiquitin receptors (Bhattacharyya et al., 2014; Sakata et al., 2012) and, given their proximity, we propose that Rpn13-bound SGTA and Rpn10-bound BAG6 can modulate the access of MLPs to the proteasome (cf. Fig. 7). This model is also supported by the finding that disrupting the interaction of SGTA with the N-terminal UBL of Bag6 reverses the ability of the exogenous protein to stabilise MLPs (Wunderley et al., 2014). Hence, the interaction of SGTA with both Rpn13 and the BAG6 complex appear to be important for its role in protein quality control (Fig. 7).

In addition to binding SGTA (this study), the C-terminal region of Rpn13 has also been shown to bind and activate UCH37 at the 19S proteasome (Bhattacharyya et al., 2014; Hamazaki et al., 2006; Yao et al., 2006). On this basis, we speculate that – following the arrival of MLPs at the proteasome – SGTA controls the access of these substrates to proteasome-associated DUBs (Fig. 7) and, thereby, influences MLP degradation (Wunderley et al., 2014), consistent with the effects on steady-state MLP levels that we observed in this study. Although we had previously suggested that proteasomal components are dispensable for the SGTA-mediated stabilisation of MLPs (Leznicki and High, 2012), it is now apparent that MLPs can be dealt with by alternate cellular quality control pathways that are – at least partially – redundant in nature (Rodrigo-Brenni et al., 2014). Interestingly, UCH37-mediated deubiquitylation can either suppress or promote polypeptide degradation in a substrate-specific manner (D'Arcy and Linder, 2012; Lee et al., 2011) and, hence, SGTA might impact on either of these potential fates for MLPs. We speculate that, in a physiological context, BAG6/SGTA-dependent cycles of substrate ubiquitylation and deubiquitylation are able to distinguish between aberrantly and correctly folded precursor proteins, thereby enhancing the fidelity of quality control (Brodsky, 2013; Wunderley et al., 2014; Zhang et al., 2013). Alternatively, SGTA binding and/or SGTA-facilitated deubiquitylation might provide a ‘rescue pathway’ for endogenous hydrophobic substrates, such as tail-anchored proteins, that might be prone to premature ubiquitylation (Ast et al., 2014; Leznicki and High, 2012; Wunderley et al., 2014). In the latter case it is noteworthy that, in addition to providing the binding site for Rpn13 (this study), the TPR domain of SGTA can also interact with Hsp70 and Hsp90 chaperones (Liou and Wang, 2005; Walczak et al., 2014), thereby providing substrates with potential access to additional quality control factors (cf. Fig. 7). Such a system would allow a putative BAG6/SGTA cycle to provide a proteasomal triage pathway, enabling aberrant precursors several attempts at productive folding/ER delivery before they are committed to degradation, and ensuring that the delivery of precursor proteins into competing pathways for maturation and degradation is carefully controlled (Fig. 7) (Leznicki and High, 2012; Wunderley et al., 2014).

## MATERIALS AND METHODS

### Materials

Standard molecular biology techniques were used to clone the variants of mouse Rpn13, human Rpn10 and human SGTA into pGEX-4T1 vector for bacterial production of GST-tagged recombinant proteins. The His Trx-SGTA fusion protein is as previously described (Leznicki et al., 2011). Plasmid pGEX-4T2- TetraUb was a kind gift of Caixia Guo and Errol Friedberg (University of Texas, Dallas, TX). For expression in mammalian cells, full-length Rpn13 and SGTA were cloned into NpFLAG-CMV2 plasmid, whereas full-length Rpn10 was in pcDNA3.1-myc. Where used, SGTA-V5, Bag6-V5 and OP91 were in pcDNA5 and OPG-TASK_85_ in pcDNA3.1 are as previously described (Leznicki and High, 2012; Wunderley et al., 2014). The K160E/R164E mutant of SGTA is defective for Hsp70 binding (Walczak et al., 2014; Xu et al., 2012), and was generated by site-directed mutagenesis and validated by DNA sequencing prior to use. The Ub-R-GFP cDNA was obtained from Addgene (plasmid number 11939) deposited by Nico Dantuma (Karolinska Institute, Sweden) (Dantuma et al., 2000), and re-cloned into pcDNA5 vector using a TOPO cloning kit (Invitrogen).

Anti-Rpn13 and anti-20S proteasome antibodies were obtained from Enzo Life Sciences, anti-FLAG M2 antibody from Sigma, anti-Myc antibody (clone 4A6) from Upstate and anti-His tag antibody from Novagen. Mouse anti-SGTA antibody (clone 47-B) was purchased from Santa Cruz Biotechnology, whereas a chicken anti-SGTA antibody was made to order. Mouse anti-tubulin antibody was a gift from Keith Gull (University of Oxford, UK). Rabbit anti-tubulin, rabbit anti-GFP, rabbit anti-PSMD1 and chicken anti-Bag6 antibodies were from Abcam, whereas mouse anti-V5 antibody was purchased from Abcam and Serotec. The monoclonal anti-opsin tag antibody has been previously described (Leznicki et al., 2010). The HEK293 cell line stably expressing the hRpn11-HTBH plasmid (HEK293^Rpn11-HTBH^) was a kind gift of Lan Huang (University of California, Irvine, CA) (Wang et al., 2007). Stable, tetracycline-inducible HeLa Flp-In T-REx cells used to generate a stable line expressing OP91 were from Stephen Taylor (University of Manchester, UK) (Tighe et al., 2008; Wunderley et al., 2014).

### Recombinant protein production and GST pull-down assay

*Escherichia coli* strain BL21 transformed with the pGEX-4T1 plasmid encoding a GST-tagged protein of interest was grown to OD_600_=0.5; expression was induced with 0.5 mM IPTG followed by overnight incubation at 16°C. Cells were harvested, lysed by sonication in lysis buffer [20 mM Tris-HCl pH 7.5, 10 mM EDTA, 5 mM EGTA, 150 mM NaCl, 0.5% (v/v) Triton X-100] and insoluble material pelleted by centrifugation (20 min, 4°C, 10,000×***g***). Pre-equilibrated Glutathione Sepharose 4B beads (GE Healthcare) were added to the supernatant, incubated for 1 h at 4°C and extensively washed with the lysis buffer. Bound GST fusion proteins were either stored on the resin at 4°C or released by using thrombin (GE Healthcare) at 24°C in 1× cleavage buffer (20 mM Tris-HCl, pH 8.4, 150 mM NaCl, 2.5 mM CaCl_2_, 1 mM DTT) overnight. Beads were then pelleted by centrifugation and thrombin was inactivated with PMSF. The interaction between purified components was followed by incubating one Glutathione-Sepharose-bound protein with a potential binding partner that had been released from its GST tag through thrombin cleavage. The His-tagged Trx-SGTA fusion protein and a K160E/R164E mutant version were expressed and purified as previously described (Leznicki et al., 2011). Binding reactions were performed in 1× incubation buffer [20 mM Tris-HCl pH 8.4, 150 mM NaCl, 2.5 mM CaCl_2_, 10% (v/v) glycerol, 1% (v/v) Triton X-100, 1 mM DTT] for 4 h at 4°C, beads washed extensively with incubation buffer, SDS sample buffer added and samples resolved by SDS-PAGE followed by western blotting.

For pull-down experiments from mammalian cell lysate, HeLa cells were transiently transfected with the indicated plasmids using GeneJuice (Merck) according to manufacturer's instruction. After 24 h cells were lysed in ice-cold lysis buffer [50 mM HEPES pH 7.5, 150 mM NaCl, 1 mM EDTA, 1 mM EGTA, 10% (v/v) glycerol, 1% (v/v) Triton X-100, 25 mM NaF, 10 mM ZnCl_2_] freshly supplemented with complete protease inhibitor cocktail (Roche) and, after a pre-clearing centrifugation step (20 min, 13,000×***g***, 4°C), the soluble fraction was incubated for 4 h at 4°C with recombinant proteins immobilised on Glutathione Sepharose resin. Beads were washed with lysis buffer, bound proteins eluted with SDS sample buffer and subjected to SDS-PAGE and western blotting.

### Additional cell culture techniques

To purify the proteasomal fraction, parental HEK293T or HEK293^Rpn11-HTBH^ cells were transfected with the indicated plasmids using GeneJuice (Merck) and then lysed 24 h post-transfection in ice-cold buffer (50 mM sodium phosphate, pH 7.5, 100 mM NaCl, 5 mM MgCl_2_, 10% (v/v) glycerol, 0.5% (v/v) NP-40) freshly supplemented with complete protease inhibitor cocktail (Roche), 5 mM ATP and 1 mM DTT. The resulting lysate was pre-cleared by centrifugation (20 min, 13,000×***g***, 4°C), and supernatants were incubated with streptavidin beads (Thermo Scientific) at 4°C overnight. The resin was washed with lysis buffer and bound proteins were eluted with SDS sample buffer and resolved by SDS-PAGE followed by western blotting. For comparison, a fraction of the total lysates, equivalent to 1% of the input used for the pull-down experiment, was analysed in parallel.

The effect of expression of isolated proteasomal ubiquitin receptors was addressed by co-transfecting HeLa cells grown in 12-well cell culture dishes with a combination of 0.5 µg substrate DNA, 0.2 µg pcDNA5-SGTA-V5 or control vector and 0.3 µg plasmid encoding the Rpn13 variant, Rpn10 or appropriate empty vector controls using Lipofectamine 2000 (Invitrogen) according to manufacturer's instruction. Cells were processed 22 h post-transfection as previously described (Leznicki and High, 2012) and results visualised by quantitative western blotting (LiCor Biosciences). The effects of overexpressing SGTA-V5 and the K160E/R164E mutant version were monitored by using a TRex Flp-In HeLa cell line stably expressing OP91 under the control of a tetracycline-inducible promoter. After transfection with plasmids expressing V5-tagged SGTA variants, or a Pex19 control, samples were grown for 24 h. Cells were then induced by treatment with 1 µg/ml tetracycline, grown for further 24 h, harvested directly into sample buffer and analysed by quantitative western blotting (cf. Wunderley et al., 2014). For the quantification of substrate levels, the relevant experiments were independently repeated at least three times, the amount of substrate quantified with Odyssey 2.1 software and plotted relative to the matched control using GraphPad Prism 4.0 software.

## Supplementary Material

Supplementary Material
