# Supplementary material for: Binding of SGTA to Rpn13 selectively modulates protein quality control
Source: J Cell Sci. 2015 Sep 1;128(17):3187–96. doi: 10.1242/jcs.165209 (PMC4582187; doi:10.1242/jcs.165209)
Supplement: Supplementary Material [file supp_128_17_3187__index.html]

Binding of SGTA to Rpn13 selectively modulates protein quality control — Supplementary Material 

# Binding of SGTA to Rpn13 selectively modulates protein quality control

## JCS165209 Supplementary Material

- Supplementary Material
