# Supplementary material for: Binding of SGTA to Rpn13 selectively modulates protein quality control
Source: J Cell Sci. 2015 Sep 1;128(17):3187–96. doi: 10.1242/jcs.165209 (PMC4582187; doi:10.1242/jcs.165209)
Supplement: Supplementary Material [file supp_jcs.165209_JCS165209supp.pdf]

Supplementary material.

SGTA binding to Rpn13 selectively modulates protein quality control

Leznicki et al. 2015.

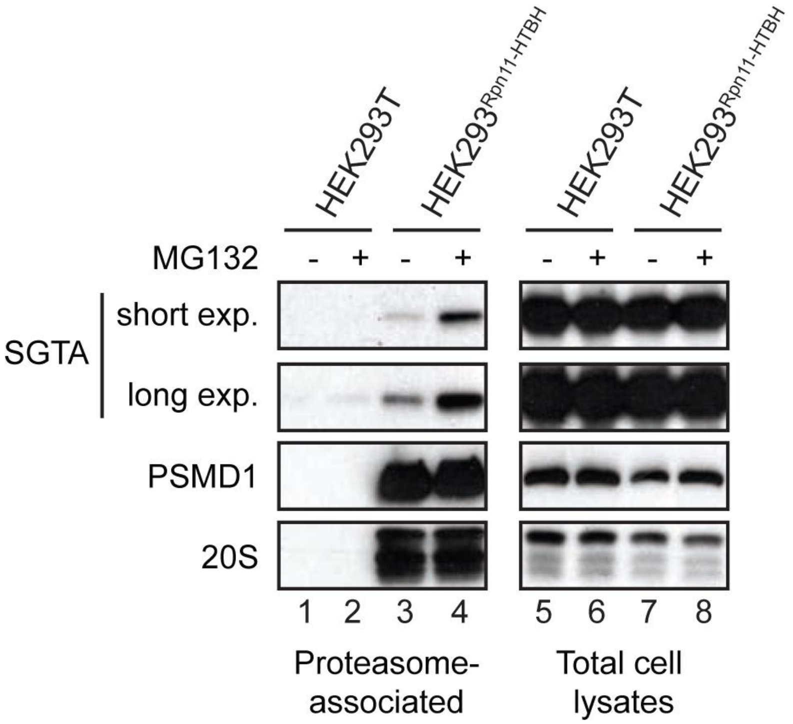

Figure S1. The association of endogenous SGTA with the proteasome

Control parental HEK293T cells, or HEK293$^{Rpn11\text{-}HTBH}$ cells that express an exogenous tagged form of Rpn11 in addition to the endogenous protein, were treated with 10 µM MG132 or DMSO (solvent control) for 16 h. Proteasomes were isolated using streptavidin beads, and the specific recovery of HTBH tagged proteasome confirmed by immunoblotting for PSMD1 (Rpn2), a 19S subunit, and components of the 20S proteasomal particle (20S). The association of endogenous SGTA with purified proteasomal fractions (lanes 1 to 4), together with the relative expression of SGTA (lanes 5 to 8), were determined by Western blotting for SGTA. Both short and long exposures of the resulting enhanced chemiluminescence signals are shown.

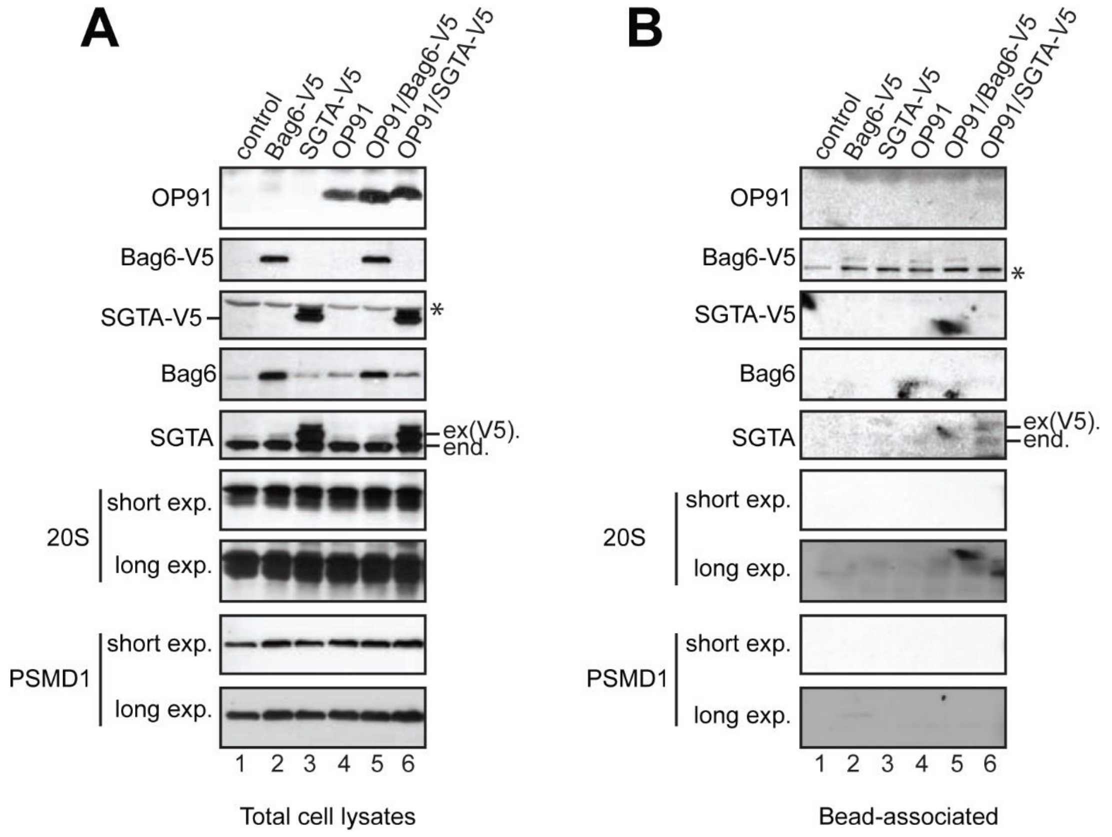

Figure S2. Specificity of proteasome isolation from HEK293$^{Rpn11\text{-}HTBH}$ cells

Parental HEK293T cells were transiently transfected with plasmids encoding the indicated proteins (lanes 2 to 6) or an empty vector control (lane 1). Total cell lysates (A) and fractions non-specifically associated with streptavidin beads (B) were analysed by Western blotting with appropriate antibodies for the presence of OP91, exogenous Bag6-V5, exogenous SGTA-V5, endogenous Bag6 and endogenous SGTA. Endogenous (end.) and exogenous (ex(V5).) SGTA are indicated. The non-specific recovery of proteasomes was determined by using antibodies against subunits of the 20S proteasome (20S) and PSMD1 (cf. Fig. 3 of main text). Results shown for OP91, V5 (Bag6), V5 (SGTA), Bag6, SGTA, 20S (short exp.) and PSMD1 (short exp.) originate from the same blots and the same exposure time as the corresponding panels from Figures 3A and 3B of the main text. Longer exposures (long exp.) of the 20S and PSMD1 signals are included to confirm the absence of any detectable proteasome recovery from control cell lysates. Non-specific, cross-reacting, species are identified by an asterisk.

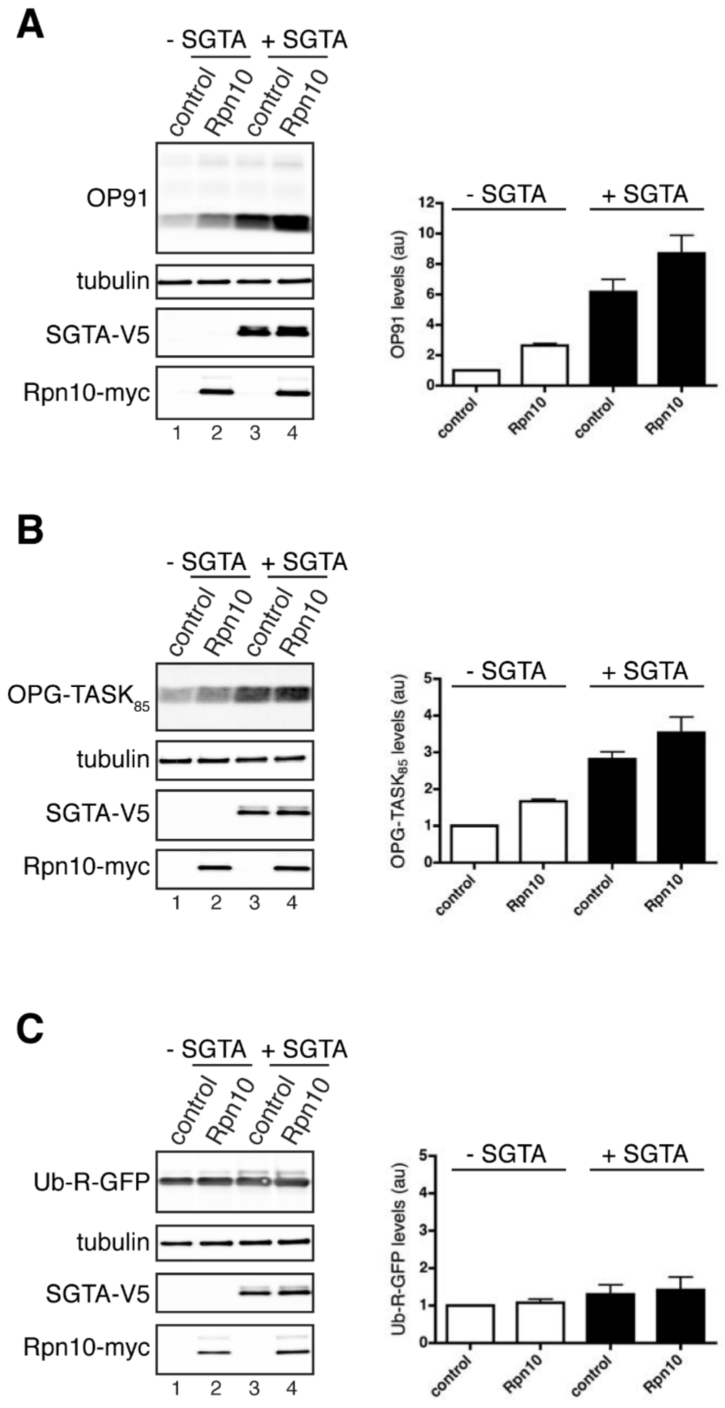

Figure S3. Overexpression of Rpn10 increases steady state MLP levels

(A-C) HeLa cells were treated as described in the legend to Figure 5 of the main text, except that the NpFLAG-CMV2 encoding variants of Rpn13 were substituted with pcDNA3.1-Rpn10-myc and the empty pcDNA3.1-myc vector used as a control in place of NpFLAG-CMV2. Overexpressed exogenous Rpn10 was visualised by Western blotting with anti-myc antibody.
